# Delayed Hemobilia: A Rare Case of Biliary Bleeding One Year After Metallic Stent Placement

**DOI:** 10.7759/cureus.47790

**Published:** 2023-10-27

**Authors:** Jaya Surya M Krishnamurthi, Shalini Verma, Janish David Johnson Bismy

**Affiliations:** 1 Internal Medicine, Madras Medical College, Chennai, IND; 2 Hospital Medicine, TidalHealth, Seaford, USA

**Keywords:** angioembolization, metallic biliary stent, sems placement, late onset biliary bleed, hepatocellular carcinoma (hcc), hemobilia, biliary stent bleed

## Abstract

Hemobilia is a rare cause of upper GI bleed. This case report discusses hemobilia caused as a delayed complication of self-expanding metallic stent (SEMS) placement in a 65-year-old male. Our patient had a history of hepatitis C and an unresectable hepatocellular carcinoma, treated with chemotherapy and radiation therapy, which caused obstructive jaundice, which in turn led to the placement of SEMS. This case highlights the challenges in managing late-onset biliary bleeding, especially in patients with underlying malignancies. Detection of bleeding in a timely manner is crucial in devising the treatment plan. Angiographic occlusion is the first line of management to stop the bleed followed by definitive surgery or stent revision. Severe complications can occur in patients with poor general health. This case report addresses the importance of monitoring the patient and the need for collaborative efforts across specialties in managing complex cases. Despite the best efforts of the medical team, this case serves as a reminder of the complex and twisted nature of medical conditions, telling us the importance of developing suitable treatment strategies for each patient’s needs and healthcare requirements.

## Introduction

There are many causes of upper gastrointestinal bleeding (UGIB). Hemobilia, or bleeding from the hepatobiliary tract, is a rare cause of acute UGIB. Bleeding from the biliary tract is usually seen after instrumentation or injury to the biliary tract or hepatic parenchyma. It can also be seen during the early postoperative period of biliary stent placement. Biliary stents are used to treat nonmalignant diseases (primary sclerosing cholangitis, biliary stricture) or malignant obstruction of the biliary tract. There are two kinds of biliary stents used in practice. Plastic biliary stents are used if the patient’s life expectancy is considered to be less than three months. For patients with distal biliary obstruction from unresectable cancer and whose expected survival is greater than three months, typically self-expanding metallic stents (SEMS) are placed. SEMS remain patent for a longer duration and provide effective drainage [1,2]. Bleeding from the biliary tract after stent placement is common in the early postoperative period. But late onset bleeding is infrequent and only a few cases are reported. Patients becoming hypotensive due to UGIB is not very common unless the bleeding is severe. The classic triad of hemobilia, which includes abdominal pain, jaundice, and gastrointestinal bleeding, is not commonly observed [3]. Here, we present a case of a 65-year-old male who presented to us with an episode of syncope and an episode of hematemesis due to bleeding from the biliary tract a year after the stent placement. In this context, our research question is: What is the effective management strategy for delayed hemobilia following SEMS placement in patients with malignancies?

## Case presentation

**Figure 1:**
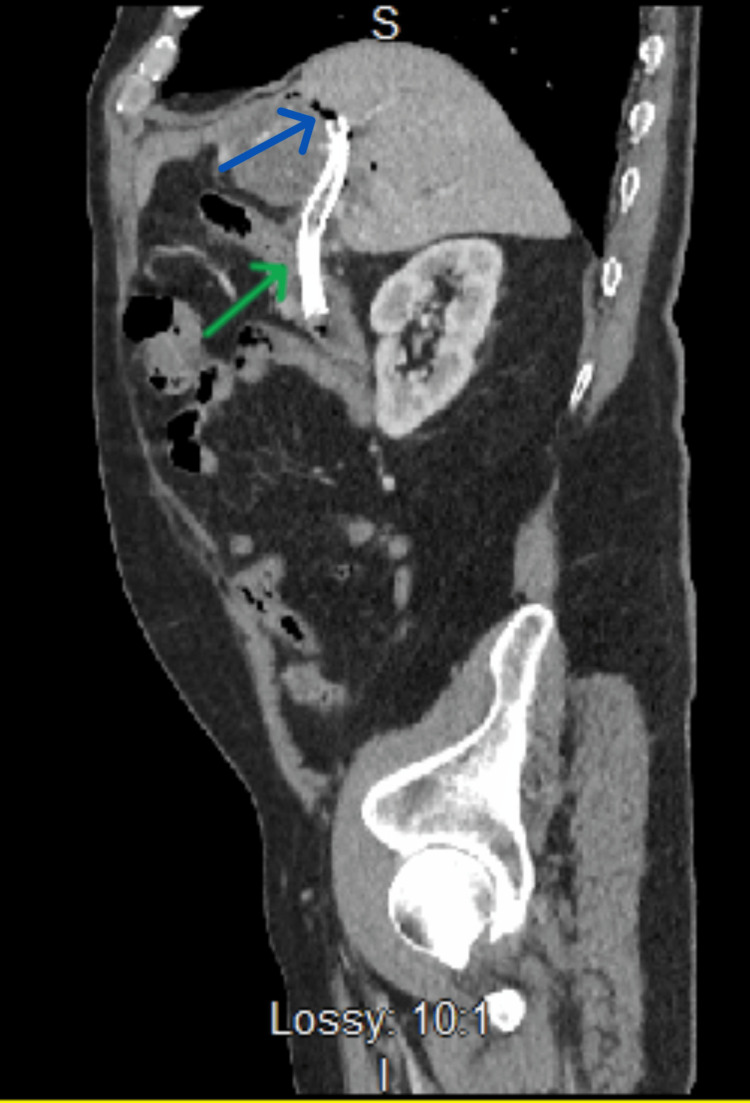
Sagittal CT image of the abdomen. There is mild pneumobilia (blue arrow) and the stent is in place (green arrow).

**Figure 2:**
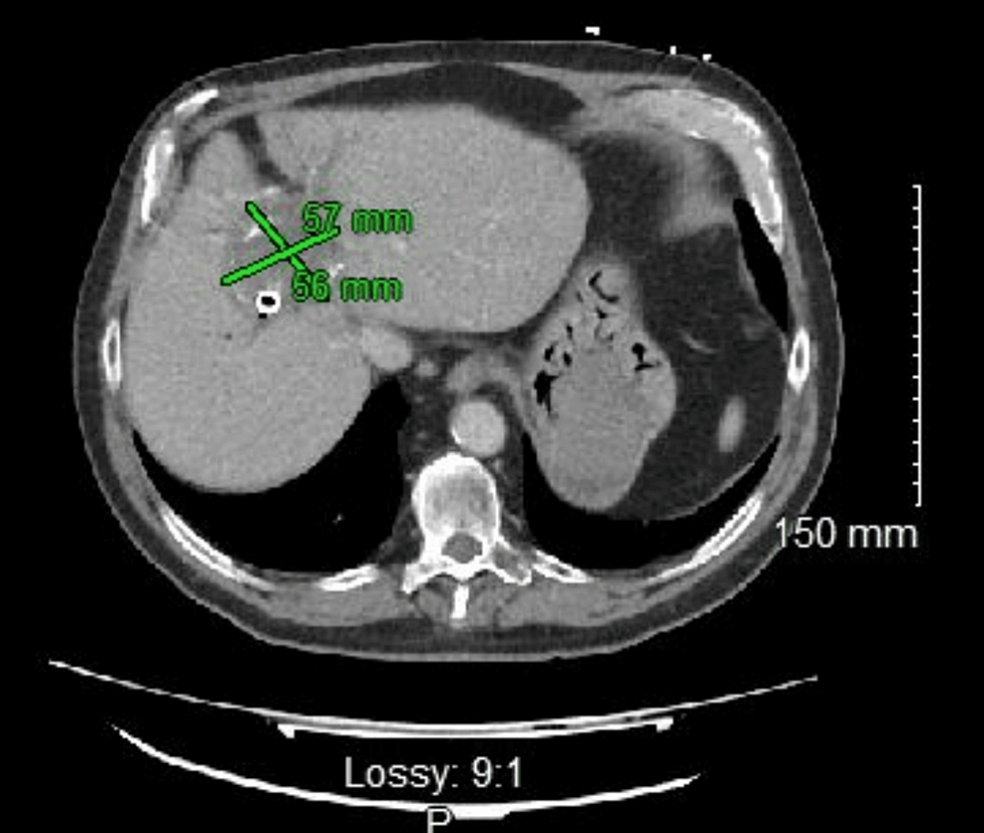
Axial CT image of the upper abdomen. There is diffuse hemobilia (green cross mark). The green cross mark measures the size of hemobilia within the liver.

A 65-year-old man presented to the emergency department with complaints of hematemesis and syncope. The patient had a history of hepatitis C and an unresectable hepatocellular carcinoma, which was diagnosed four years before the presentation. The patient was treated with chemotherapy and radiation therapy until one year before the presentation for which he had a good response. Then the patient developed obstructive jaundice due to stricture likely caused by radiation therapy and had subsequently undergone biliary stent placement. During that procedure, a SEMS had been inserted into the common bile duct. There was no relevant family history. The patient presented to the emergency department with an apparent history of lightheadedness, followed by an episode of syncope. On the way to the emergency department with the paramedics, the patient had an episode of hematemesis. The patient also had an episode of melena. On examination, the patient was diaphoretic, afebrile, and tachycardic (heart rate of 100 bpm). His blood pressure was 100/70 mmHg and his respiratory rate was 12 breaths per minute. Laboratory findings were as follows: hemoglobin of 11.7 g/dL (his baseline was 16.4-16.8 g/dL); white blood cell count of 9800/µL (normal: 3600-11200/µL); total bilirubin was 0.98 mg/dL (normal: 0.3-1.2 mg/dL); aspartate aminotransferase/alanine aminotransferase was 43/66 IU/L (normal: 15-41/17-63 IU/L); alkaline phosphatase of 166 IU/L (normal: 56-155 IU/L); platelet count of 350 x 10^3^/μl; international normalized ratio of 1.3; and creatinine of 1.31 mg/dL. Contrast-enhanced CT showed mild pneumobilia and metallic stent, which was in place (Figure 1). In the axial view, hemobilia could be appreciated (Figure 2). The CT also showed a stable mass of size 5.7 cm in the superior right hepatic lobe consistent with hepatocellular carcinoma. The patient's Child-Pugh score was 6 points and the Model for End-Stage Liver Disease-sodium (MELD Na) (UNOS/OPTN) score was 15 points at the time of presentation.

**Figure 3:**
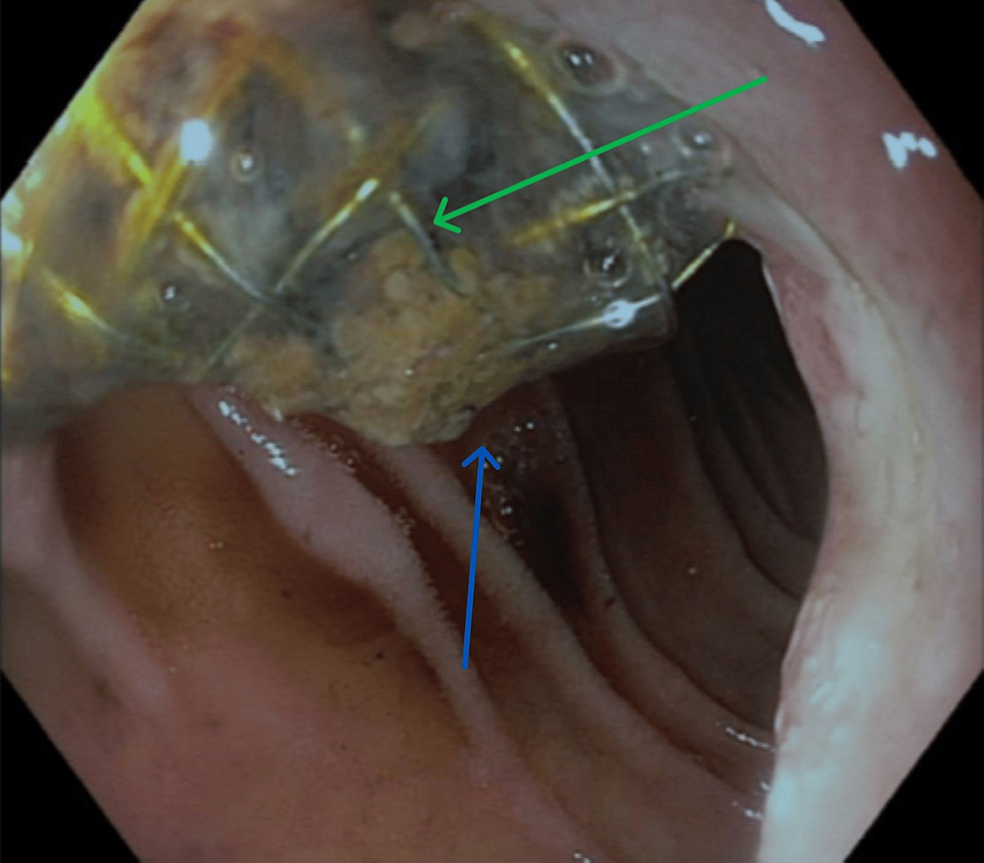
Upper gastrointestinal endoscopy revealed metallic biliary stent from the papilla (green arrow) and a small amount of bile with blood trace from the papilla (blue arrow).

**Figure 4:**
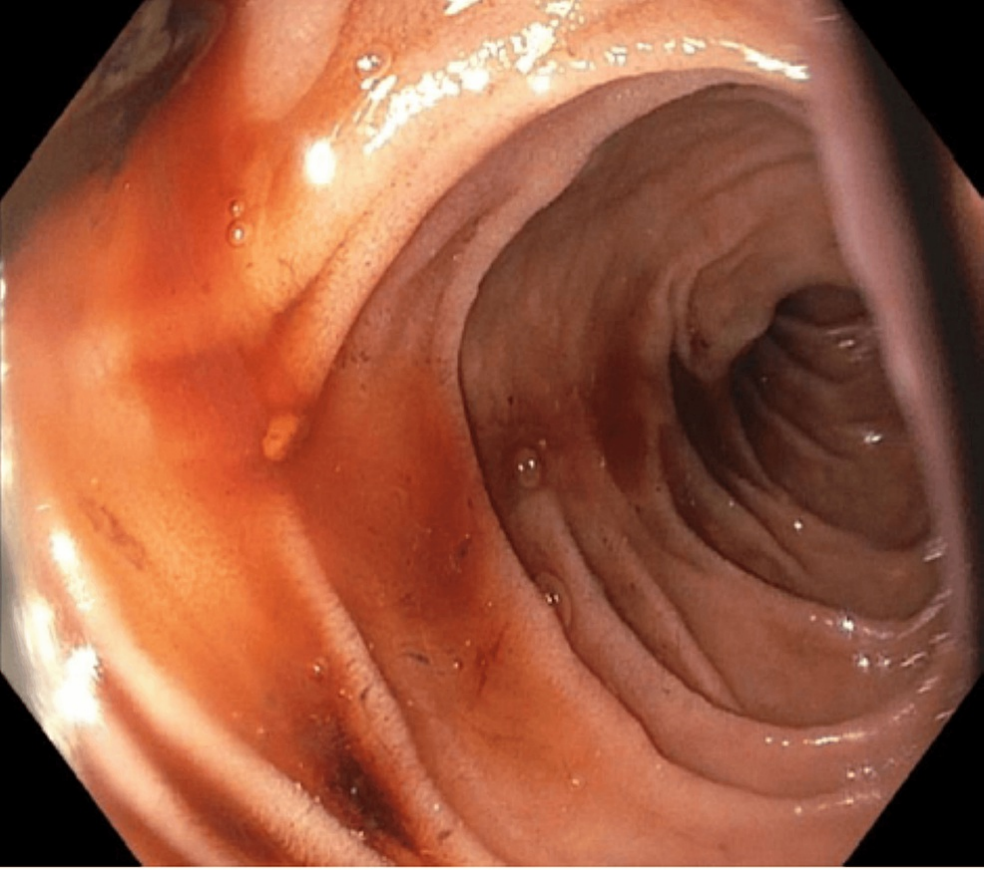
Upper gastrointestinal endoscopy revealed blood seeping throughout the third part of the duodenum.

The patient was admitted due to the large drop in hemoglobin from his baseline of 16 mg/dL to 11.7 mg/dL. The next day the patient’s hemoglobin further dropped to 8.9 mg/dL. The patient was taken to the endoscopy suite and upper esophagogastroduodenoscopy (EGD) was performed, which revealed bleeding from the biliary tract papilla (Figures 3, 4). There was mild antral gastritis with no evidence of esophageal varices. A few hours after the EGD, the patient became unresponsive and decompensated. CPR was done and after obtaining the return of spontaneous circulation, the patient was shifted to the ICU and intubated. The patient’s hemoglobin further dropped to 6.9 mg/dL and two units of packed red blood were transfused for which the patient developed a febrile rash. The patient’s hemoglobin increased to 8.6 mg/dL but dropped to 7 mg/dL the same evening. The patient was shifted to the interventional radiology suite and selective hepatic artery embolization was done with Gelfoam, a liquid embolic agent.

Extubation was performed the same day but the patient developed fever spikes, tachycardia, and increased lactate levels suggesting an inflammatory response. Antibiotics were started. Despite initial stabilization, the patient’s condition deteriorated rapidly with a drop in consciousness and developed bradycardia. The patient went into cardiac arrest and despite extensive resuscitation efforts, he could not be revived.

## Discussion

Biliary obstruction in malignancy is usually caused by a tumor mass compressing the biliary tract. Rarely can it be caused due to stricture caused by radiation therapy. SEMS have played an important role in the treatment of bile duct obstruction caused by primary or metastatic malignancy [4-6]. The most common early complication of percutaneous stent placement is transient hemobilia from hepatic parenchymal bleeding that spontaneously subsides with conservative management [7-9]. Major delayed complications of biliary stents are biliary occlusion and cholangitis. However, delayed life-threatening biliary bleeds are rarely seen.

The next step after suspecting hemobilia is to perform a computed tomography angiography (CTA) of the abdomen and upper EGD. The diagnosis of hemobilia is often overlooked if the papilla is not carefully examined endoscopically. In our case, bile with a trace of blood could be seen from the papilla and blood could be seen pooled in the duodenum. There have been biliary bleeding cases reported from hepatic pseudoaneurysm in patients with biliary stents [10]. The CTA did not reveal any active bleeding or pseudoaneurysm in this patient. In this case, we suspect the bleeding was caused by constant erosion of the artery wall by the radial force of the metallic stent.

When a bleed is suspected, the recommended first-line treatment is angiographic occlusion. This approach is advised to control hemobilia and stabilize the patient while preparing them for elective and definitive surgery, which is either a stent revision or a biliary duct repair [3]. This procedure was done in our patient, after which he was stable initially, but deteriorated later. The reason for deterioration could be due to one of the following reasons: persistent or recurrent hemorrhage, complications related to angiographic occlusion procedure such as sepsis or postembolization syndrome, and inadequate hemodynamic support. The rate of severe complications is greater in patients with malignancies as their general condition will be often poor [11]. This patient had increased lactate levels, tachycardia, and fever spikes all pointing toward an inflammatory response. The cause of death in this patient could be due to hypoperfusion from either septic shock or hypovolemia.

In this case, we highlighted two things. One is the need for early detection and timely intervention and the other is the role of angiographic occlusion as the first-line treatment for suspected hemobilia. It is important to recognize that patients with malignancies are at a higher risk of severe complications. Adequate hemodynamic support and periodic monitoring are essential, especially in this population.

Despite our efforts, this patient's condition deteriorated, which emphasizes the complexity of managing such cases. By sharing this case and our experience, we hope that healthcare professionals can remain alert, consider various possibilities and alternative strategies, and coordinate with different specialties to improve outcomes for patients facing these complex situations.

## Conclusions

Though a rare late postoperative complication following biliary stent placement, late-onset hemobilia can cause the patient’s condition to deteriorate at a fast pace. The management poses a challenge when associated with comorbidities, especially immunocompromised states like malignancy. Early detection and timely management with angiographic occlusion and antibiotic coverage while hemodynamically stabilizing the patient is crucial. Continued care and keen monitoring are essential during the post-embolization period. After achieving hemostasis and stabilizing the patient, the patient can be planned for elective and definitive surgeries like stent revision. We conclude by emphasizing that physicians should be aware of the possibility of a biliary bleed post stent placement even after one year.
